# Cholinergic receptor pathways involved in apoptosis, cell proliferation and neuronal differentiation

**DOI:** 10.1186/1478-811X-7-20

**Published:** 2009-08-27

**Authors:** Rodrigo R Resende, Avishek Adhikari

**Affiliations:** 1 Department of Physics, Institute of Exact Sciences, Federal University of Minas Gerais, Belo Horizonte, MG, 31270-901, Brazil; 2 Institute of Learning and Research Santa Casa of BH (IEPSC – BH), Belo Horizonte, Brazil; 3 Department of Biological Sciences, Columbia University, New York, NY, 10027, USA

## Abstract

Acetylcholine (ACh) has been shown to modulate neuronal differentiation during early development. Both muscarinic and nicotinic acetylcholine receptors (AChRs) regulate a wide variety of physiological responses, including apoptosis, cellular proliferation and neuronal differentiation. However, the intracellular mechanisms underlying these effects of AChR signaling are not fully understood. It is known that activation of AChRs increase cellular proliferation and neurogenesis and that regulation of intracellular calcium through AChRs may underlie the many functions of ACh. Intriguingly, activation of diverse signaling molecules such as Ras-mitogen-activated protein kinase, phosphatidylinositol 3-kinase-Akt, protein kinase C and c-Src is modulated by AChRs. Here we discuss the roles of ACh in neuronal differentiation, cell proliferation and apoptosis. We also discuss the pathways involved in these processes, as well as the effects of novel endogenous AChRs agonists and strategies to enhance neuronal-differentiation of stem and neural progenitor cells. Further understanding of the intracellular mechanisms underlying AChR signaling may provide insights for novel therapeutic strategies, as abnormal AChR activity is present in many diseases.

## Introduction

Acetylcholine (ACh) is an ancient signaling molecule, [1] and is present in both prokaryotes and eukaryotes [2-4]. Although ACh has been extensively studied for its role as a neurotransmitter, it also has autocrine functions [5] in diverse cell types. ACh has been shown to promote cytoskeleton organization, cellular proliferation, differentiation and apoptosis [2-4,6-8] throughout development [2,3,9]. Intriguingly nAChR signaling pathways have been preserved throughout evolution [10], suggesting that they have critical functions. We shall attempt to discuss the physiology of ACh as well as ACh's relevant downstream pathways in apoptosis, cell proliferation and neuronal differentiation of embryonic stem cells.

Interestingly, nicotinic receptors are expressed in undifferentiated and differentiating cells, [8,11-13] suggesting that ACh-mediated signaling between neuronal and non-neuronal cells may influence cell fate [8,11,12,14]. Supporting this idea, ACh has been shown to modulate neuronal cell differentiation during development [15,16]. Moreover, transfecting a non-neuronal cell line such as a neuroblastoma with choline acetyltransferase induces expression of neuronal markers, muscarinic receptors and production of ACh [14]. Lastly, ACh also regulates cell proliferation [17] and apoptosis [18]. These and other findings marked the beginning of a new field: the role of nAChRs in the development and progression of cancer and in stem cell physiology.

## Nicotinic ACh receptors

ACh receptors can be nicotinic (nAChRs), which are ion channels, or G protein-coupled (GPCR) muscarinic receptors (mAChRs). In the central nervous system, nAChRs have been shown to regulate diverse processes such as neurotransmitter release and cellular excitability. Nicotinic receptors also influence physiologic processes such as arousal, sleep, fatigue, anxiety, pain processing, hunger and various higher cognitive functions. [19-22].

### nAChRs structure and function

nAChRs are multisubunit proteins of neuromuscular and neuronal origins. These receptors form ligand-gated ion channels that mediate synaptic transmission both in the neuromuscular junction and between neurons. Since various neuronal nAChR subunits exist, nAChRs can be formed by different combinations of subunits. [23]. Nicotinic receptors of different compositions exhibit different specificities for various ligands and are thereby pharmacologically distinguishable. For example, the elapid alpha-neurotoxins that block activation of nAChRs at the neuromuscular junction do not block activation of other neuronal nAChR subtypes [24].

A functional nAChR consists of five subunits which may be different (certain combinations of α1–9 and β1–4, γ, δ, ε subunits) or identical (α7–9) i.e. subunits [25]. All subunits have a similar structure with one extended extracellular domain (N-terminal), four transmembrane domains (M1–M4), one intracellular domain of variable length which joins M3 and M4 domains and one small extracellular C-terminal domain [26]. The binding site for ACh and other agonists is located on the N-terminal extracellular domain at the boundary between α and non-α subunits. In heteromeric neuronal receptors the α and β subunits contribute to the binding site The amino acid sequence analysis of various subunits shows that nicotinic receptors can be divided into three sub-classes. The first family includes α-bungarotoxin-sensitive muscle-type heteromeric receptors, typically found in skeletal muscle and fish electrical organs, with (α1)_2_β1γδ and (α1)_2_β1γε pentameric structures in fetal and adult form, respectively. The second family includes nAChRs consisting of α-bungarotoxin-insensitive, heteromeric subunits. These receptors have various combinations of α2, α3, α4 and α6 with β2, β4, α5 and β3 subunits. The third family includes α-bungarotoxin-binding nicotinic neuronal receptors consisting of five identical subunits (α7, α8 or α9) [19].

Neuronal nAChRs are expressed in the autonomic nervous system ganglia, and in the CNS, in post- pre and extra synaptic locations. The α7 nAChR subtype is highly expressed in regions of the brain involved in learning and memory, such as the hippocampus and the neocortex [27]. This subtype has a particularly high permeability for calcium ions, increases glutamatergic neurotransmission, and modulates neuronal plasticity by influencing the growth of axons [28].

Studies on the structure, functions and pharmacology of nAChRs neuronal receptors are necessary because these receptors are involved in a large number of nervous system diseases (for review see Clementi and Adlkofer Special Issue on "nicotinic neuronal receptors" 2000).

## Muscarinic ACh receptors

### mAChRs structure and function

Muscarinic receptors are members of the G Protein-coupled receptors (GPCRs), and are composed of a family of five receptor subtypes (M1, M2, M3, M4 and M5). These receptors are widely distributed on multiple organs and tissues and are critical to the maintenance of central and peripheral cholinergic neurotransmission. The distribution of these receptor subtypes in the brain and other organs has been extensively studied. M1 is the predominant subtype found in the cerebral cortex and is involved in the control of cognitive functions. M2 is the main subtype in the heart and is believed to play a role in the control of heart rate. M3 is involved in gastrointestinal and urinary tract functioning as well as sweating. M4 is present in the brain and may have a role in locomotion. Lastly, M5, also present in the brain, modulates certain functions of the central nervous system associated with the dopaminergic system, such as dopamine release in the nucleus accumbens following mesopontine stimulation in mice [29]. The M5 subtype is also important for brain-stimulation reward [30], opiate reward [31], latent inhibition learning and amphetamine-induced locomotion [32-34].

As mAChRs are involved in such a wide array of processes, it is not surprising that muscarinic signaling has been shown to be abnormal in many diseases, such as overactive bladder [35,36], chronic obstructive pulmonary disease [36,37], neurodegenerative disease as Alzheimer's disease [38], dementia [39], Sjogren's disease [40], vascular dementia [41] and others. Consequently, there is considerable interest in finding pharmacological agents to selectively modulate each receptor subtype. Agonists such as muscarine and pilocarpine and antagonists such as atropine have been known for over a century; however, these drugs do not target specific subtypes. Unfortunately, little progress has been made in the discovery of subtype-selective compounds making it challenging to study specific functions of individual receptor subtypes. The clinical utility of classic muscarinic antagonists such as atropine is limited due to the high incidence of both peripheral and central adverse effects such as tachycardia, blurred vision, dryness of mouth, constipation, dementia, etc. Atropine derivatives such as ipratropium bromide are better tolerated but most of them lack of selectivity for specific muscarinic receptor subtypes. [42-44]. Thus, the search for subtype selective muscarinic drugs remains an active area of research.

Muscarinic subtypes are coupled to different GPCRs, leading to activation of distinct downstream pathways. M1, M3 e M5 are G-protein coupled receptors (subunit α of the G_q/11 _family), while M2 and M4 subtypes are coupled to the α subunit of G_i _and G_o_. Consequently different second messenger dependent-pathways being activated by each muscarinic subtype. For example, phospholipase Cβ (PLC) is activated by M1, M3 and M5, while adenilate cyclase is activated by M2 and M4 subtypes [45]. Processes modulated by pathways involving these G-proteins include smooth muscle contraction (through M2 and M3 receptors), stimulation of glandular secretion (M3 receptors) and inhibition of cardiac voltage-dependent calcium channels (M2 subtypes) [45].

## Expression and function of n- and mAChRs in embryonic cells

ACh [46] is present in the brain prior to axonogenesis and synaptogenesis, suggesting that it may mediate non-classical signaling. Furthermore, muscarinic receptors are widely expressed in embryonic cells [8,13,47-54], and have been shown to regulate neuronal cell proliferation and differentiation [47,50,51]. In neuronal progenitor cells, muscarinic receptor expression also occurs prior to the onset of synaptogenesis and neurotransmission [9,55], indicating that ACh may act via a local autocrine loop in the embryo. In early development, M2 receptors are expressed in the dorsal root ganglia neurons, as well as in non-neural cells such as Schwann cells, where they control sensory neuronal differentiation and axonal growth [56]. Intriguingly, muscarinic receptors are also expressed in primary and metastatic tumor cells in which ACh also acts in an autocrine fashion [57]. As discussed above, expression of muscarinic receptors is an embryonic trait. The expression of these receptors in tumor cells [8,13,58-63] likely arises from reactivation of embryonic genes during malignant growth [52]. Proliferation due to mAChR activation has been reported in many tumor cells. For example, activation of muscarinic M1, M3 or M5 muscarinic receptors (but not M2 or M4 receptors) induces foci of transformation in 3T3 cells [64,65]. Furthermore, activation of the Gq_α11_-coupled muscarinic receptors in various cell lines [59,66,67] or the α7 nicotinic subtype in P19 embryonic carcinoma cells [13,68,69] induces growth and proliferation. These effects of muscarinic receptor activation depend on the cellular phenotype [70,71]. Activation of M3 induces proliferation in human colon cancer cell lines and prostate carcinoma cells [72]. In contrast, activation of endogenous M3 inhibits DNA synthesis in several small cell lung carcinoma cell lines [72]. These contradictory responses also occur in cells transfected with mAChRs, as transfected M3 mAChRs has been reported to both inhibit and stimulate proliferation [73-75]. In cells deprived of trophic factors, muscarinic M3 receptor activation elicits anti-proliferative signals via activation of the small GTP-binding protein, Rac1 [72,76,77]. However, muscarinic agonists can also inhibit apoptosis. Pretreatment of tumor cell lines with muscarinic agonists inhibits apoptosis induced by DNA damage [78-81]. Moreover, agonists for M1, M3 and M5 subtypes showed a protective response against apoptosis in Chinese hamster ovary cells transfected with these muscarinic receptors [82]. This effect occurs via a mechanism independent of Ca^2+^/PLC signaling that may involve upregulation of the anti-apoptopic protein Bcl-2 [6,83]. It is unclear whether muscarinic receptor activation is related to tumor growth and stem cell proliferation [8,84-87]. In addition to morphogenesis, non-neuronal muscarinic receptors also control cell migration, as M3, M4 and M5 subtypes were shown to control cell migration by facilitating fibronectin-induced movement [58,88-90]. Contraction and aggregation of cells in embryonic tissue is also induced by muscarinic receptor activation [91]. In this way, mAChRs could modulate cellular movement during morphogenesis [92,93].

## Apoptotic signaling pathways

### Apoptotic pathways associated with mAChRs

**Figure 1:**
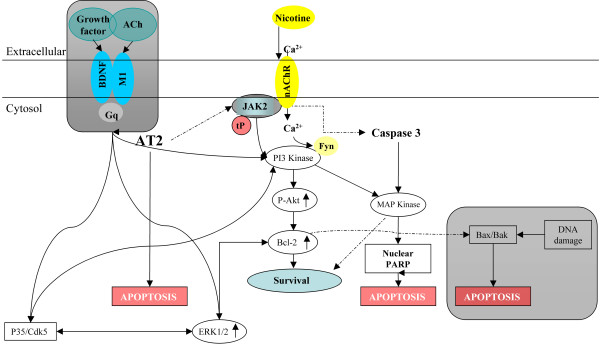
**Diagram depicting apoptotic pathways modulated by nAChRs and mAChRs in neuronal progenitor cells**. See text for a better description.

Many G-protein-coupled receptors protect cells from apoptosis induced by growth factors, DNA damage or cellular stress. Among those receptors, mAChRs have been shown to be protective in many cell lines and primary cell cultures [8,78-80,94,95]. These reports raise the possibility that damage to cholinergic pathways might contribute to the development of neurodegenerative disorders, such as Alzheimer and Huntington. In some neurodegenerative disorders losses of neuronal survival stimuli occur, leading to cell death. It is known that mAChRs inhibit apoptosis through activation of PI3-kinase (phosphatidylinositol-3-OH kinase) and its downstream targets, protein kinase B (PKB)/Akt and MAPK/ERK (Figure 1) [96]. These kinases activate pro-survival pathways in diverse cell types [96], including neurons [97,98]. Previous work [77,80,99-101] has demonstrated that Akt can be activated effectively through G_q _coupled to M1 and M3, and G_i _coupled to M2. Activation of Akt occurs through βγ complexes and the α, Gα_q _and Gα_i _subunits of Gs proteins. The cell survival effects mediated by mAChRs are partially blocked by inhibitors of the PI3-kinase MAPK/ERK pathways [99]. Interestingly, Lindenboim et al., [78], have demonstrated that the protective effects of ACh in undifferentiated and neuronal PC12 cells require M1 receptors. In this condition, expression of M2 receptors decreases DNA synthesis, arresting the cell cycle at S and G_2_/M. Furthermore, activation of M1 and M3 receptors inhibits caspase 2 and 3, and this effect has been shown to be independent of PI3-kinase and MAPK/ERK pathways [77,79,102]. Previous reports have described cholinergic signaling in progenitor and tumoral cells. Gutkind and collaborators [73] showed how mAChRs exert oncogenic control in neuronal cell growth by regulating signaling of extracellular-1/2/MAP. Similar results were also obtained in non-neuronal cell types [103-105]. Although it is unclear if mAChR activation is related to oncogenic progression, there are reports suggesting that inhibition of mAChRs decrease cell proliferation [8,106]. In line with these results, previous work showed that apoptosis can be induced in Chinese hamster ovary cells transfected with the M3 receptor and exposed to toxic substances [83]. This effect might be related to caspase action, considering that M3 receptor activation did not prevent cell death. This process may be specific for G_q/11 _coupled muscarinic subtypes, specifically M1, M3 and M5 [79,83]. However, the use of a version of M3 truncated at its carboxyl end revealed that this protection is not mediated by PLC or by phosphorylation of its receptor. Moreover, these pathways are not involved in activation of ERK and JNK, so presumably, the anti-apoptotic actions of mAChRs are not mediated by these proteins, but through its own C terminal domain, which is rich in basic residues [83]. The mechanism through which mAChRs mediate cell survival is dependent on transcription of the anti-apoptotic protein Bcl-2, which can be induced by mAChRs [83]. This protective feature of mAChRs may be a conserved among other G-protein coupled receptors.

By targeting two signaling pathways, the MAPK/ERK pathway [107,108] and the PI3-kinase signaling pathway [109], it would be possible to gain new insights on the intracellular signaling pathways through which mAChRs inhibit apoptosis. It has been suggested that these pathways inhibit caspases [77,79,102]. To this end, it was shown that PKB/Akt, the downstream target of PI3-kinase, can inhibit both caspase-9, [110-112] and the pro-apoptotic protein Bad [112-115]. However, PKB/Akt can also be activated by other pathways. These are mediated by protein kinase A (PKA), calcium/calmodulin-dependent kinase and Bad [114,116-119]. Interestingly, inhibitors of the PI3-kinase or MAPK/ERK pathways could not suppress the muscarinic effect on caspases, although all were able to inhibit the muscarinic-dependent phosphorylation of PKB/Akt and MAPK/ERK. Furthermore, previous studies in PC12 cells have shown that these inhibitors block survival effects and ERK and PI3-kinase signaling [120,121]. It has been suggested that the MAPK/ERK and PI3-kinase pathways are not essential for mediating the muscarinic effect on caspase activity in PC12 cells [79], but rather that this effect is mediated by an unidentified pathway. This finding is in line with recent studies showing that in some cases the survival effects of growth factors and cytokine receptors are mediated by pathways independent of both MAPK/ERK and PI3-kinase. For example, neither the survival effect of nerve growth factor (NGF) on sympathetic neurons [122] or rat-1/MycER cells transfected with TrkA [123], nor that of granulocyte/macrophage colony-stimulating factor on MC cells, is mediated by the PI3-kinase pathway [124]. Moreover, the MAPK/ERK and the PI3-kinase pathways are not essential for the survival effect of NGF on apoptosis induced by ceramide in PC12 cells [121]. However, the PI3-K/Akt pathway has an established role in NGF-promoted cell survival [125]. Activation of PI3-K by NGF initiates a cascade involving Akt, which leads to the phosphorylation and inhibition of the pro-apoptotic protein Bad and activation of the pro-survival inhibitor κB kinase α (IKKα) [116,126]. Blockade of the PI3-K/Akt pathway by a dominant negative Akt mutant or treatment of cells with PI3-K inhibitors reveal that activation of this signaling pathway is required for NGF-promoted cell survival [127]. However, it was shown that pertussis toxin did not inhibit Akt phosphorylation. This finding is consistent with the previous observation of pertussis toxin having a more profound inhibitory effect during the early phase of NGF-induced Erk1/2 activation [128]. Upon activation, the TrkA receptor activates the PI3-K pathway, leading to activation of Akt in sympathetic neurons [125]. Previous studies have shown that TrkA forms complexes with GRK2 and GAIP/GIPC (GAIP-interacting protein, C terminus) [128,129], thereby providing a bridge to link TrkA and G protein signaling pathways. Furthermore, there is prior evidence pointing to a functional association between pertussis toxin-sensitive G proteins and growth factor receptors such as the insulin receptor tyrosine kinase [130]. In addition, some pertussis toxin-sensitive growth factor-induced responses have been reported. For example, insulin-like growth factor 1 has been shown to activate G_i _and release Gβγ subunits [131,132], while TrkA is able to utilize G_i/o _to stimulate Erk1/2 [128,133]. Increasing evidence shows that GPCRs often cooperate with RTKs (receptor tyrosine kinases) in the regulation of numerous signal transduction pathways [134-136]. More recently, a report has demonstrated that NGF and lysophosphatidate receptor signaling systems can interact to promote G protein-mediated activation of the Erk pathway [133]. These observations are consistent with the notion that TrkA can utilize pertussis toxin-sensitive G_i/o _proteins to activate Akt, thereby inhibiting Bad and stimulating the NFκB regulator, IKK, via G_αγ _activation [137,138] to allow cell survival through the M3 subtype [83,139,140].

One candidate for mediating the muscarinic effect on caspases is sphingosine-1-phosphate, which was shown to play an important role in the survival effect of NGF on PC12 cells [141]. Interestingly, sphingosine-1-phosphate can be induced by the M2 muscarinic receptor [142]. Intriguingly, inhibition of the PI3-kinase pathway partially attenuates the muscarinic survival effect on the viability of the cells but not on caspase inhibition. One possible explanation is that serum-deprived cells can die via both caspase-dependent and -independent pathways, as shown in some apoptotic paradigms such as Bax-induced cell death in the presence of caspase inhibitors [143-145]. Despite the fact that the caspase-dependent pathway seems to play a major role in the death of serum-deprived PC12 cells, it is possible that once this pathway is inhibited, the caspase-independent pathway has a major role. However, one cannot exclude the possibility that there are unknown caspases which are activated and involved in apoptosis induced by trophic-factor-deprivation and that these caspases are inhibited by the muscarinic receptor in a different mechanism than that used to inhibit the DEVDase caspases and caspase-2. It was shown that NGF withdrawal from differentiated PC12 cells induces expression of FasL, which in turn may contribute to the apoptotic process via activation of the CD95 receptor [146,147]. In this case, it is still possible that muscarinic receptors will inhibit the activation of caspase-8, the caspase which is directly activated when CD95 is activated [80,115,148] by a PI3-kinase-dependent mechanism as was shown for CD3 activation in Fas-treated Th2-type cells [80,115]. In some systems, one signaling pathway appears to be sufficient for mediating survival induced by trophic agents such as NGF (PI3-kinase) and *N*-acetylcysteine (ERK) [109,120]. However, in other systems, the survival effect may require the combined action of several signaling pathways. For example, insulin-like growth factor-1 inhibited apoptosis in differentiated PC12 cells requires both PI3-kinase and MAPK/ERK signaling pathways [127,149,150]. It has been suggested that the muscarinic survival effect could be mediated by the combined effect of at least two different pathways. One pathway may lead to caspase inhibition and be independent of PI3-kinase and ERK signaling. This pathway could be mediated by the G_i/o_-coupled receptors. The other pathway would act through G_q _and may involve the PI3-kinase pathway, and could promote survival by a mechanism that does not affect caspases and that could be mediated by the G_q_-coupled receptors.

Previous reports showed that muscarinic receptor stimulation leads to activation of the Rho family of small G-proteins [151,152]. This pathway can lead to activation of Rho kinase [153] and is critical for the protective capacity of muscarinic receptors. It has been demonstrated that excitatory receptor agonist-induced Rho activation is Ca^2+^-dependent [154]. The ability of mAChRs to activate SRF-mediated gene transcription and the involvement of G protein subunits in SRF activation were investigated in Jurkat T cells. It has been shown that Gα_q_-coupled M1, but not Gα_i/o_-coupled M2 receptors can activate SRF through a RhoA-mediated pathway [73,155]. In contrast, M3 mAChR failed to activate SRF even though M1 and M3 are thought to induce similar signaling pathways that involve Gα_q/11_. Yet, Gα_q _coupling of both M1 and M3 remained intact, as revealed by a robust calcium response in Jurkat T cells. Moreover, use of the chimeric Gα protein construct Gα_iq5_, which allowed the M2 receptor to signal along Gα_q/11_-mediated pathways, restored the Ca^2+ ^response for M2, but not SRF activation. This suggests either that the activation of SRF through M1 involves a Gα_q/11_-independent pathway or that Gα_q/11 _is insufficient in Jurkat T cells. The inhibition of M1-SRF signaling by co-transfection with the Gα_q/11 _suppressors, RGS2 and RGS4, indicated that Gα_q/11 _did play a role in M1-SRF activation, but it appeared to be insufficient *per se*. However, an increase of M1-SRF signaling inhibition was observed when intracellular calcium was decreased.

### Apoptotic pathways associated with nAChRs

**Figure 2:**
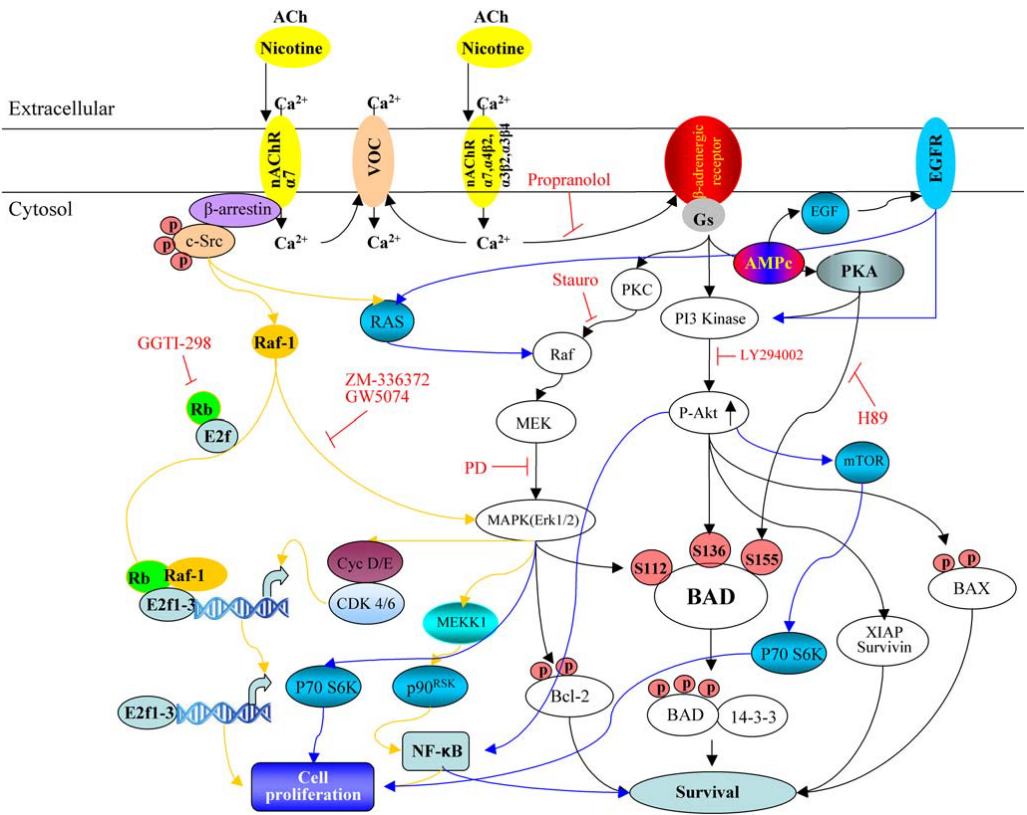
**Diagram depicting proliferative and survival signaling pathways in cells**. *In vitro*, the homomeric and heteromeric nAChRs jointly stimulate the indicated signaling cascades. Yellow arrows indicate proliferative pathways triggered by nAChRs. This activation triggers the MAP kinase pathway, leading to DNA synthesis. Sustained mitogenic signaling induces to S-phase entry. Black arrows indicate nAChR survival and proliferation pathways triggered by intracellular calcium increases involving indirect activation of β-adrenergic receptor signaling, which in turn, induces activation of epidermal growth factor (EGF) receptor leading to the cascade indicated by blue arrows. α7 nAChR and heteromeric α-βnAChRs are activated by their agonists. Influx of Ca^2+ ^and other cations through the nAChRs and voltage-gated Ca^2+ ^channels trigger the release of adrenaline and noradrenalin. Adenylyl cyclase activation downstream of β-adrenergic receptors induce the cyclic AMP-protein kinase A (PKA)-CREB (cAMP response element-binding protein) pathway, transactivates epidermal growth factor receptor (EGFR) and induces the release of EGF, and perhaps another growth factors. The responsiveness of this pathway is enhanced by α7 nAChR-mediated activation of Ras through β-arrestin-dependent SRC signaling. In turn, the EGFR activates the Akt pathway and its downstream effectors, X-linked inhibitor of apoptosis protein (XIAP)-survivin and nuclear factor-κB (NF-κB).

Nicotinic receptors are expressed in neural and non-neuronal tissues; however, in the latter their function is not clear. Although nAChRs are primarily known for their action as ligand-gated ion channels transducing action potentials across synapses, they may have other actions as well, such as cell-to-cell communications in various non-neuronal tissues controling important cell functions such as proliferation, adhesion, migration, secretion, survival and apoptosis in an autocrinal, justacrinal and paracrinal manner [22]. Interestingly, nicotinic receptors in neurons protect against cell death in some settings [8,12,68,81]. In neurons, the α7 nicotinic receptor activates PI3-kinase through a src-family kinase, activating the anti-apoptotic kinase AKT [156]. One pathway involved in AKT signaling involves phosphorylation of the forkhead transcription factor FKHRL1, causing its retention in the cytoplasm associated with 14-3-3. This in turn blocks expression of the apoptotic protein fas [157]. The PI3-kinase/AKT pathway protects a broad range of neurons against apoptotic cell death and may block apoptosis triggered by beta-amyloid fragments, which contribute to progression of Alzheimer's disease. If so, nicotinic agents may prove useful in the treatment of this and other neurodegenerative conditions. Interestingly, removal of extracellular Ca^2+ ^suppressed Akt phosphorylation induced by nicotine. It was shown that an inhibitor of Src tyrosine kinase also reduced Akt phosphorylation. In addition, PI3-K and Fyn are physically associated with α7 nicotinic receptors. Therefore, nicotinic receptor stimulation might lead to phosphorylation of Akt through Fyn [157-159]. The α7 subtype can mobilize Ca^2+ ^from ryanodine-sensitive intracellular stores and promote cell survival [8,13]. This intracellular Ca^2+ ^mobilization can lead to BDNF-induced Cdk5-mediated neuroprotection through increased Bcl-2 expression. (Figures 1 and 2). Nicotine has also been shown to regulate the Bcl-2 family of proteins. For example, nicotine induces phosphorylation of Bcl-2 leading to protection of human small cell lung carcinoma cells against cisplatin-induced apoptosis [160,161]. Moreover, nAChRs heterodimers containing α3 and α4 mediate their apoptotic activity in normal human bronchial epithelial cells through Akt [162]. Lastly nicotine also phosphorylates downstream targets of Akt, such as mTOR, FKHR, elf-4, GSK3b, tuberin and S6K [162].

Nicotine can also promote anti-apoptotic effects through activation of PKC, PKA and NF-κB, and downregulation of the tumor suppressor p53 [160,161]. Nicotine-induced NF-κB phosphorylation promotes the phosphorylation of the apoptotic protein Bad (Bcl-2 antagonist of cell death), which becomes inactivated and prevents cell death. Other pathways underlying the anti-apoptotic effects of nicotine include the MEK and PI3K pathways [163]. It was previously shown that ERKs, AKT, and PKA could function as a Bad Ser^112^, Ser^136^, or Ser^155 ^kinase, respectively [164-167]. One interesting study demonstrated that nicotine-induced Bad phosphorylation is mediated by β-adrenergic receptors [163] (for a review see [168]), and that nicotine can also induce phosphorylation of Bax (another Bcl-2 antagonist of cell death) through PKCζ, thereby inactivating Bax and suppressing cell death [169,170].

The protective effects of nicotine have been studied in NSCLC and PC12 cell lines, as well as in other experimental systems [171-176]. Nicotine can protect A549 NSCLC cells against apoptosis induced by anticancer drugs through the upregulation of XIAP and survivin in a α3-nAChR-dependent manner [171]. Furthermore, administration of nicotine in the CNS can stimulate release of neurotransmitters [177,178] and neurotrophic factors, such as basic fibroblast growth factor (bFGF or FGF-2) and brain-derived neurotrophic factor (BDNF)[179]. In addition, nicotine exposure can lead to elevated cellular cAMP levels [180]. It was demonstrated that nicotine attenuates both arachidonic acid-induced caspase activation and apoptosis of spinal cord neurons [181]. However, controversy exists on the specific nAChR subunit responsible for the anti-apoptotic effects of nicotine. The α7 and the α4β2 subtypes may play the most significant role in the central nervous system [180,182,183]. Data from some laboratories indicate that phosphorylation of ERK1/2 in nicotine-treated neurons can be specifically prevented by pre-exposure to the α7 blocker α-bugarotoxin, but not dihydro-β-erythroidine (an antagonist of the β2 subunit) [172,178,181], indicating an important role for the α7 receptor in nicotine-mediated neuroprotection. On the other hand, studies in tumor and tumoral stem cells have implicated the dihydro-β-erythroidine-sensitive α3 and α4 receptors. This finding implies that the proliferative effects (mediated by α7 nAChR) and pro-survival effects (mediated by α3 or α4 nAChR) of nicotine are mediated by two distinct classes of receptors [8,13,162,171]. These data demonstrate that during differentiation there are changes in nAChR activity and function. This could be due to β-adrenergic receptor activation through α7 nAChR, which has been found to mediate the anti-apoptotic effects of nicotine in some cell lines [184-186] (for a more detailed discussion see [168]). Such discrepancies can be partially explained by the pleiotropic nature of nAChR subunit inhibitors. It is also possible that the anti-apoptotic effects of nicotine are mediated by different nAChR subunits in a tissue-specific manner. These possibilities underscore the need for further studies to identify the nAChR subunits responsible for the anti-apoptotic effects of nicotine.

It is known that ERK1/2 signaling can participate in the neuroprotective effects of nicotine through a variety of different mechanisms. Previous reports showed that ERK2 can increase expression of bcl-2 and inhibit apoptosis [187]. In addition, the neuroprotective effects of ERK1 and ERK2 may be related to activation of a variety of transcription factors, which in turn can regulate transcription of neurotrophic factors, leading to overexpression of "survival" genes and enhanced neuronal viability. Among the transcription factors that are involved in the ERK-mediated cellular survival are Elk1, nuclear factor-κB (NF-κB), and cAMP response element (CRE)-binding factor (CREB) [188-190]. A role for NF-κB in neuronal survival has been suggested [191]. In addition, recent evidence indicates that CREB has a crucial role in regulation of cell viability [192], as it is required to induce transcription of BDNF [193]. Elk1 functions as a nuclear transcriptional activator through the interaction with the serum response element (SRE), which is present in the promoter of many immediate early genes [194]. Among others, Elk1 is involved in regulation of expression of FGF-2 [195,196].

## Signaling pathways of mAChRs involved in proliferation and neuronal differentiation

**Figure 3:**
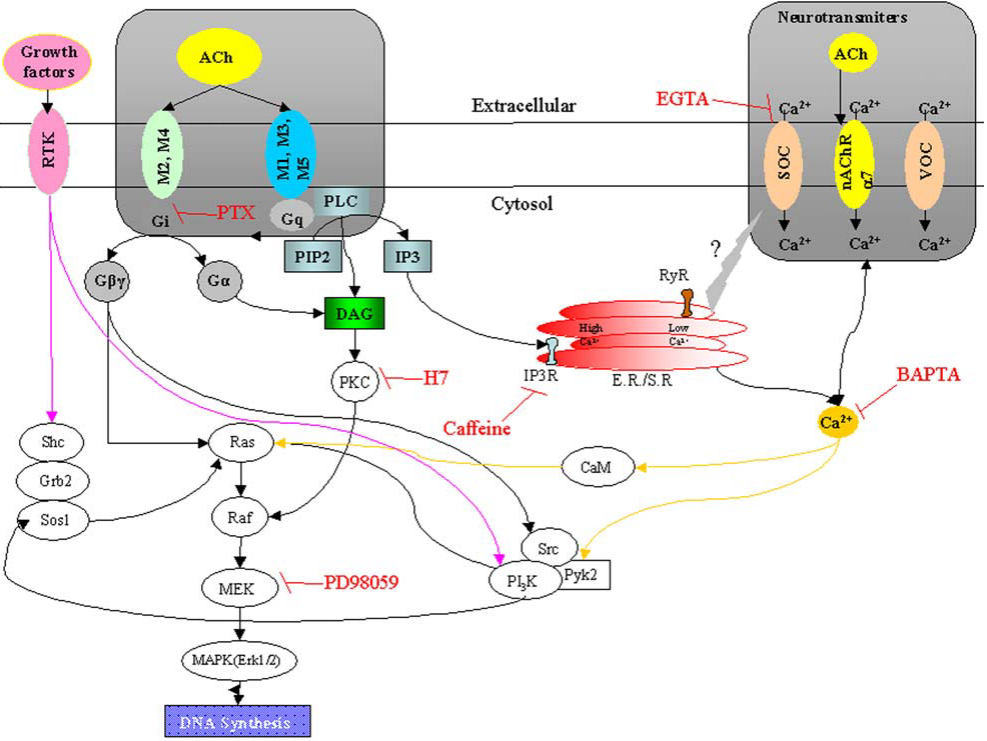
**Muscarinic and nicotinic receptor-coupled signal transduction pathways mediating MAPK activity and proliferation**. Both Erk1/2 activity and cell proliferation are activated by cholinergic (ACh) stimulation of mAChRs. ACh binds to M3, leading to a G_q_-protein-mediated activation of PLC, which hydrolyses PIP2 to IP3 and DAG, subsequently mobilizing Ca^2+ ^from organellar stores, leading to activation of PKC. Both ACh-induced MAPK activity and proliferation are reduced by the PKC inhibitor H7, indicating that PKC activity appears to be one of the upstream events critical to MAPK activation. mAChR stimulation induces increases in [Ca^2+^]_i _via both Ca^2+ ^influx and mobilization from intracellular stores. Muscarinic stimulation of MAPK activity and proliferation is prevented both by BAPTA-AM and EGTA, demonstrating that elevation of [Ca^2+^]_i _is essential, and may stimulate Pyk2 phosphorylation and activate the MAPK. Muscarinic stimulation of MAPK activity is effectively eliminated by the MAPK kinase (MEK) inhibitor PD98059. M2 and M4 may provide parallel pathways to MAPK activation via pertussis toxin-sensitive G_i_-proteins and βγ subunits. ERKI/II (MAPK) can serve as a convergence site for multiple extracellular signals known to induce plasticity in mature neurons. The best documented activation of the MAPK cascade occurs via ligand binding to RTK. Activation of RTK recruits the Shc-Grb2-SOS1 complex, which in turn activates Ras. Ras induces MAPK activation via an evolutionarily conserved pathway, which includes Raf, MEK (MAPKK), and ERKI/II (MAPK) (*MAPK cascade*). ERKI/II is known to have both cytoplasmic and nuclear targets and can translocate to the nucleus to modulate transcription in neurons. The block of proliferation induction by the MEK inhibitor PD98059 suggests that MAPK plays a role in the induction phase of proliferation. MAPK can also be activated in neural progenitor cells via nAChRs which increase [Ca^2+^]_i_, and may modulate the MAPK cascade via activation of a Ca^2+^-dependent tyrosine kinase (*PYK2*) or calmodulin (*CaM*).

**Figure 4:**
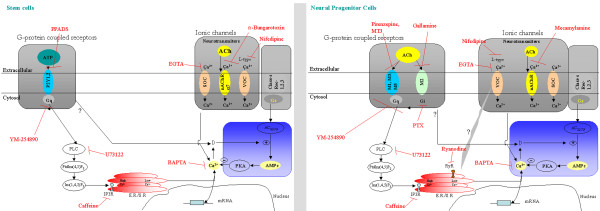
**Calcium signaling pathways in stem cells and neural progenitor cells**. Left panel represents a embryonic or adult stem cells and right panel the neuronal progenitor cells. Ca^2+ ^signaling depends on the increase of the intracellular Ca^2+ ^levels [Ca^2+^]_i_, derived from extracellular calcium (Ca^2+^)_o _sources or intracellular stores of the endoplasmic reticulum (ER Ca^2+^). It can enter through calcium channels operated by voltage (voltage-operated Ca^2+ ^channels, VOCCs) in excitable cells such as neurons and muscular cells, or through calcium channels operated by receptors (receptor-operated Ca^2+ ^channels, ROCs) in response to neurotransmitters. SOC's (store-operated Ca^2+ ^channels, SOCs), open when internal Ca^2+ ^stores are empty, and are generally present in non-excitable cells. Calcium from the ER is released by two types of channels, Inositol 1,4,5-trisphosphate (IP3) channels and ryanodine channels. The first is present in both neural progenitor and stem cells, while the latter is expressed only in nural progenitor cells. IP3 is generated by the action of the enzyme PLC in phosphatidylinositol 4,5-bisphosphate (PIP2). IP3 acts on receptors in the endoplasmic reticulum, promoting the release of Ca^2+ ^from ER stores. IP3PIP2.

Previous studies have demonstrated that proliferation and differentiation of neuronal precursor cells can be modulated by mAChR signaling [8,13,197,198]. The mechanism involved initially amplifies mAChR and nAChR signals, inducing calcium influx, which in turn activates MAPK-dependent pathways [8,13,199]. Transient calcium increases induced by ACh independently of MAPk activation has been shown to be necessary for differentiation and proliferation, as muscarinic antagonists and calcium chelating agents block these effects (Figures 3 and 4) [8,13].

Activation of M2 and M3 receptors has been shown to increase proliferation of tumoral cells in a dose-dependent manner. Cellular proliferation induced by the M3 subtype is mediated by production of inositol triphosphate, [8,66] and nitric oxide [for a review see [200]], while the effects of the M2 subtype were dependent on concomitant activation of M1, promoting the release of E2 prostaglandin and arginase catabolism. These events are related to tumoral cell growth [66], and inhibition of caspases [79,83].

In murine mammary adenocarcionoma cells, the M3 subtype is the most highly expressed muscarinic receptor. Stimulation of M3 receptors activates adenilate cyclase, phospholipase A_2 _(PLA_2_), IP3 and diacylglicerol (DAG) through PLC [201]. Each of these molecules in turn activates different pathways. DAG activates protein kinase C (PKC), while IP3 induces release of calcium from intracellular stores. It is known that both pathways regulate MAPK and ERK signaling. Free intracellular Ca^2+ ^can modulate MAPK/ERK either through Ca^2+^-dependent protein tyrosine kinase (PYK2) [202] or by Ca^2+^/calmodulin kinase (Ca^2+^/CaM) [203]. PKC isoforms are also known to regulate MAPK/ERK through Raf-1 [204] or through transactivation of the epidermal growth factor receptor (EGFR) mediated by the Src/PYK2 complex (Figures 2 and 3) [205].

Recent evidence suggests that activation of MAPK/ERK through GPCRs occurs through PKC-dependent and -independent mechanisms, depending on the receptor activated and on the cell type [206,207]. It is known that activation of MAPK/ERK GPCR agonists mediate cell proliferation [55,208], and that pathways involving cholinergic receptors seem to depend on the cell growth [8,74].

Stimulation of mAChRs promotes an increase in [Ca^2+^]_i _and induces phosphorylation of MAPK/ERK in MCF-7 human breast cancer cells [105]. Activation of this pathway increases protein synthesis and cell proliferation through MAPK kinase, besides inducing DNA synthesis in neuronal progenitor cells during early neurogenesis [209]. Inhibition of PLC or incubation of cells in a calcium free medium did not alter MAPK/ERK phosphorylation; however, this phosphorylation can also be induced through treatment with phorbol 12-myristate acetate (PMA), a PKC activator. Activation of MAPK/ERK was not affected by PKC modulation or by its inhibition. Interestingly, phosphorylation of MAPK/ERK by mAChRs could be blocked by a PKC-ζ (a miroystoilated pseudo substrate of PKC) inhibitor and by high doses of staurosporine (a relatively non-selective protein kinase inhibitor). This pathway involves PI3-K and tyrosine kinases, such as Src, and Erk 1/2 [105,209]. Cells in the neuroepithelial ventricular zone of the embryonic rat cortex also express the M2 receptor. The presence of M2 induces cell proliferation and accelerates neuronal differentiation.

Adrenergic receptors can transform fibroblasts when actively mutated [210]. Interestingly, transformation by mAChRs was ligand-dependent [211]. Furthermore, some viruses encode constitutively active GPCRs linked to cell proliferation (for a review see [212,213]), suggesting that signals initiated by GPCRs can be mitogenic.

MAPKs target numerous cellular proteins and transcription factors involved in cell growth and differentiation [214-216]. It is known that GPCRs activate MAPK through the small GTP-binding protein, p21Ras [217]. How p21Ras is activated is still controversial; however, it is likely that transactivation of EGFR upon stimulation of GPCRs participates in p21Ras activation [218,219]. Indeed, several types of GPCRs including thrombin, endothelin, and angiotensin II receptors have been shown to transactivate EGFR, leading to MAPK activation [220,221].

The vast majority of the currently described pathways leading to ERK stimulation have been considered as linear. While GPCRs coupled with Gi-protein activate the p21Ras-ERK pathway through the βγ subunit and PI-3 kinase, GPCRs with Gq-protein activate it in a PKC-dependent manner [222,223]. However, Blaukat et al. [223] recently showed that GPCRs mediate ERK activation through cooperation of Gi and Gq, suggesting that multiple G-proteins could act in concert to attain full activation of p21Ras-ERK pathway. Muscarinic receptors in many cells have been shown to activate ERK by carbachol, and this is not altered by treatment with pertussis toxin, indicating that Gq-, but not Gi-protein, may be involved in ERK activation [61,224-229]. Muscarinic receptor activation by carbachol rapidly and transiently stimulates ERK1/2 phosphorylation in many cells in a time- and dose-dependent manner [61,226-229], as observed in various cell lines [230,231]. It was shown that the inhibition of PLC led to a total blockade of Ca^2+ ^mobilization induced by AChRs agonists [13,232]. A role of Ca^2+ ^in this pathway is also supported by the finding that an increase in intracellular Ca^2+ ^caused by thapsigargin [8,13,105,233] is sufficient to induce ERK phosphorylation up to levels similar to those induced by AChRs agonists. The mechanisms by which intracellular Ca^2+ ^stimulates the phosphorylation of ERK1/2 are complex and appear to be dependent on the nature of mAChR subtype coupling to heterotrimeric G proteins. Intracellular Ca^2+ ^can modulate the MAPK cascade, via activation of the monomeric G-protein p21^ras ^[234-236], through two convergent mechanisms; one through the calcium-dependent tyrosine kinase (PYK2) and the other mediated by calmodulin [228,231,237]. In T_84 _colon epithelial cells, which express endogenous M3 mAChR subtypes, increases in [Ca^2+^]_i _in response to carbachol activate signaling mechanisms involving calmodulin-, PYK2-, and p60^src^-mediated transactivation of the EGF receptor [238]. Besides Ca^2+^, the other downstream pathway induced after PLC activation is the PKC transduction cascade. It was demonstrated that the direct activation of PKC, by the phorbol ester PMA, was sufficient to increase the phosphorylation of ERK1/2 reaching levels similar to those induced by carbachol in FRT cells [105]. However, the carbachol-induced ERK1/2 phosphorylation was not mediated by PKC. These data indicate that the mAChR-induced ERK phosphorylation is mediated by a Ca^2+^-dependent but PKC-independent mechanism.

Although some data suggest that intracellular Ca^2+ ^partly mediates the activation of ERK1 and ERK2, other intracellular signaling pathways may be involved in the MAPK/ERK activation in undifferentiated cells. Activation of MAPK by GPCRs, including mAChRs, involves phosphorylation of one or more proteins, such as p125^FAK^, p130^cas^, or paxillin [239]. Moreover, the Src family of protein tyrosine kinases has been implicated in mAChR-induced ERK activation in different cell lines [240-242]. Further studies are needed to determine the connection between activation of these protein tyrosine kinases and the downstream effects of mAChR after G protein activation.

It has been suggested that carbachol's effects on ERK1 and ERK2 phosphorylation were probably mediated through the activation of protein tyrosine kinases. Furthermore, it has been demonstrated that carbachol-induced ERK activation is dependent on the activity of cytoplasmatic Src-like tyrosine kinase family, since pharmacological inhibition of the Src family of tyrosine kinases with specific PP2 blocks the carbachol-induced MAPK/ERK activation [55]. The Src family of tyrosine kinases has been implicated in the ERK activation by various GPCRs agonists. Recent data suggests that activation of Src tyrosine kinases may lead to the phosphorylation of the adaptor protein Shc and the recruitment of Grb/Sos complex to the plasma membrane, resulting in the activation of the ERK pathway [223,243].

## Signaling pathways of nAChRs involved in proliferation and neuronal differentiation

Previous reports have shown that nAChRs are expressed in non-neuronal cells within the nervous system [244,245], embryonic stem cells [8,13], neural stem cells [197], and embryonic tissues [246].

Microglia express α7 nAChRs [247], and stimulation of α7 nAChRs promotes anti-inflammatory pathways and blunts the response of migroglia to lipopolysaccaride [247], suggesting that nAChRs may have a role in controlling localized brain inflammation. Nicotinic receptors are also expressed on O2A oligodendrocyte precursors, but are not detectable after induction of differentiation, indicating that nAChR expression is developmentally controlled in these cells [248]. Although the physiological functions of nAChRs in O2A oligodendrocyte precursors are not understood, data suggest that activation of nAChRs might control migration, survival and differentiation in these cells [248].

It has been found that in non-neuronal tissues nicotine induces the secretion of growth factors like bFGF, TGF-α, VEGF, and PDGF [249] Nicotine also upregulates expression of the calpain family of proteins [250] as well as COX-2 and VEGFR-2 [251], activating the Raf/MAPK kinase/ERK pathway [252]. Since nAChRs do not have intrinsic tyrosine kinase activity [22], the molecular mechanisms underlying its effects on proliferation remain unclear. It was demonstrated that nicotine-mediated induction of cell proliferation involves recruitment of β-arrestin, which facilitates the activation of Src. This in turn leads to binding of Raf-1 kinase to Rb, leading to cell cycle entry [253]. Dasgupta and coworkers demonstrated that human non-small cell lung cancer (NSCLC) tumor tissues had high levels of Rb-Raf-1 complexes in tumors relative to adjacent normal lung tissue, suggesting that perhaps the Rb-Raf-1 pathway contributes to the genesis of these tumors. Furthermore, chromatin IP (ChIP) analysis of human NSCLC tumor samples demonstrated increased recruitment of E2F1 and Raf-1 to proliferative promoters like cdc6 and cdc25A. These results suggest that binding of β-arrestin to nAChRs is an early and critical event in the initiation of nicotine-induced mitogenesis. The subsequent steps resemble growth factor-induced cell proliferation, as they include activation of Src, association of Rb to Raf-1, inactivation of Rb, and enhanced recruitment of E2F1 and Raf-1 to promoters of genes that induce proliferation [253]. These events are likely to contribute to the growth and progression of tumoral cells.

## Therapeutic uses of cholinergic receptor modulators in stem cell specification

### *In vivo *proliferation, differentiation, and genetic modification of neural stem cell progeny

As previously demonstrated [8,13], AChRs have different roles on proliferation in embryonic and neural stem cells. In embryonic cells, nAChRs decrease proliferation. Conversely, neural stem cells and their progeny can be induced to proliferate *in vivo *by administering α7 agonists [13]. To initiate neuronal differentiation, agonists for the Gα_i_-coupled mAChRs, such as the M2 subtype, could be used for treatment. These pharmacological agents include any substance that acts through AChR activation or through pathways activated by them. The examples described here to modulate proliferation, differentiation, and genetic modification of neural stem cells *in vitro *can be adapted to *in vivo *techniques. Such *in vivo *manipulation and modification of these cells allows cells lost due to injury or disease to be endogenously replaced. This would abolish the need for transplanting foreign cells into a patient. Additionally, cells can be modified or genetically engineered *in vivo *so that they express various biological agents useful in the treatment of neurological disorders. However, fine control of muscarinic signaling requires compounds that selectively modulate specific muscarinic receptor subtypes. Unfortunately, such drugs have not been discovered yet. M1 muscarinic agonists such as arecoline have also been found to be weak agonists of M2 and M3 subtypes, and are not very effective in treating cognitive impairment, most likely because of dose-limiting side effects [254,255]. Selective muscarinic agonists for M5 and M2 subtypes could be used both as pharmacological tools and as therapeutic agents [8], as M5 and M2 mediate most of the muscarinic [Ca^2+^]_i_-response and seem to control proliferation and differentiation induction, respectively.

Treatment with nicotinic receptor agonists also has therapeutic potential, similarly to muscarinic agonists. However, nAChR agonists which bind the same site as ACh are not a viable solution, for ACh not only activates, but also blocks receptor activity through desensitization [256] and uncompetitive blockade [for review see [257]]. Furthermore, prolonged activation appears to induce a long-lasting inactivation. Therefore, agonists of ACh may reduce or enhance receptor activation. In nAChRs, desensitization generally limits the duration of current during agonist application [258]. However, positive allosteric modulators can enhance the efficacy of agonists at nicotinic receptors. It is believed that such compounds would be useful for treatment of conditions associated with decreased nicotinic transmission. In a therapeutic setting, these compounds could restore normal interneuronal communication without affecting the temporal profile of activation. In addition, they would not produce long-term inactivation, contrary to prolonged application of an agonist.

A naturally existing allosteric modulator of nicotinic transmission is the CGRP (Calcitonin Gene Related Peptide) neuropeptide [259,260]. Previous reports show that CGRP blocks nAChRs competitively. It is noteworthy to point out that this effect is not mediated by conventional G-protein-coupling [261], as it was demonstrated that this peptide's activity is contained within the 1–7 N-terminal fragment. Similarly to the native CGRP, CGRP 1–7 shows a rapidly developing, competitive antagonism which is readily reversible after washout [261]. Intriguingly, some CGRP fragments quickly and reversibly enhance responses mediated by the activation of native neuronal nAChRs [262]. Mutant versions of the CGRP peptide fragment can be used as neuronal nAChRs enhancers, as in the absence of nAChR activation these peptides were inactive [262].

The CGRP 1–6 peptide did not modify the muscle-type nicotinic receptor responses, indicating its selectivity for neuronal receptors. This finding suggests that certain peptide derivatives shorter than CGRP 1–7 exert an unusual action, involving an apparently competitive modulation of the agonist-binding site. CGRP 1–6 and its derivatives may be used for the treatment of symptoms of neurological diseases associated with functional deficits of nAChRs and may be used as stem cell neuronal differentiation enhancers.

Interestingly, nicotine has been shown to protect cells from apoptosis induced by anticancer drugs. The acquisition of drug resistance is a considerable challenge in cancer therapy, and nAChR antagonists could be potentially used in combination with established chemotherapeutic drugs to enhance the therapeutic response to chemotherapy. The bioactivity of nAChR antagonists, however, has yet to be tested in animal models. Carefully designed animal studies are essential to investigate the potential side effects of nAChR antagonists on the brain, central nervous system, immune cells and muscle cells, all of which express high levels of nicotinic receptors.

The study of the roles of nAChRs in development and progression of cancer and stem cells differentiation provides novel opportunities for the prevention and therapy of cancer and degenerative disorders. However, it is important to consider that vital cell and organ functions are regulated by these receptors. Antagonists for α7 nAChR may successfully block cancer cells and promote proliferation of stem cells without cytotoxicity to normal control cells *in vitro*, but *in vivo *it would induce adverse effects on the control of inflammatory reactions and the regulation of respiratory and cardiovascular functions, and may also lead to psychiatric symptoms [22,168,263].

Blockers of Ca^2+ ^channels are known for their anti-proliferative properties and might be an alternative because they can desensitize the hyperactive α7 nAChR (Figure 1). However, experimental findings appear to be divergent and depend on the cell type and mode of administration used. Among the calcium channel inhibitors, L- and T-type calcium blockers, were reported to inhibit neuronal differentiation [12,264-266], but have limited effect on other cell types. Mibefradil, a selective blocker of T-type channels, has significant anti-proliferative action in various cell types *in vitro *as well as *in vivo *[267,268]. The non-selective calcium blocker amlodipine is also a very effective protector of neuronal cells [269-271]. Calcium channel blockers induce a rapid decrease in intracellular Ca^2+^, even in cells lacking depolarization-induced calcium flux [272,273]. These observations suggest that amlodipine inhibits cell proliferation through intracellular signaling pathways rather than through inhibition of Ca^2+ ^entry channels.

Another possible pharmacological treatment is the use of SluRP1, a drug that reduces the responsiveness of α7nAChR to agonists. SluRP1 may have an effect similar to that of voltage-gated Ca^2+ ^channels blockers [274]. However, any attempt to prevent or treat cancer by targeting nAChRs must be based on the identification of molecular markers. The goal of this strategy is the restoration of balance between stimulatory and inhibitory signaling and not complete blockade of a given pathway.

Nicotinic signaling in non-neuronal cells has huge implications for cell fate and survival. Research in nAChR signaling networks will be especially relevant to stem cell production in a large scale and cancer treatment. Future studies will need to define both the function of different nAChR subtypes in non-neuronal cells and the downstream signaling pathways that underlie the proliferative and anti-apoptotic activities of nicotine.

Similarly to nicotinic signaling, the mechanisms that underlie the pro-mitogenic effects of muscarinic receptor stimulation have not yet been studied in detail. However, several intracellular signaling pathways that regulate the synergistic mitogenic interaction of other GPCR agonists with growth factors in neural progenitor cells have been identified (Figures 1 and 3). These pathways are not the same for every GPCR agonist The GPCRs M1 and M3 increase EGF-induced proliferation through a pathway involving Gβγ, phosphatidylinositol-3-kinase, Akt and PKC [105,238]. Muscarinic stimulation of MAPK activity and proliferation is prevented both by the intracellular Ca^2+ ^chelator BAPTA-AM and by a reduction in extracellular Ca^2+ ^with EGTA. This suggests that increases in intracellular calcium are essential, and may stimulate Pyk2 phosphorylation and then activate the MAPK signaling pathway [238]. PKC also regulates p42/p44 MAP kinase activation by muscarinic receptor agonists in neuronal progenitor cells [55].

## Concluding remarks

There is a growing body of evidence indicating that nicotinic and muscarinic receptors play important roles in stem cell differentiation and physiology. The disruption of developmental patterns and of normal function is often correlated with pathological conditions. Therefore, the controlled manipulation of ACh function may lead to novel therapies. Strikingly, studies have revealed that drugs currently used to treat disorders such as Alzheimer's disease and depression, increase adult neurogenesis, which may be the mechanism mediating the activity of these drugs. However, some of these studies are controversial, and remain to be confirmed. Hence, the role of neurogenesis in treating central nervous system disorders, as well as the effects of drugs on embryonic and adult stem cells' neuronal differentiation remain areas of active research. Discriminating specific contributions of nAChR and mAChR signaling for the control of phenotypic features as specialized structural and functional behaviors is a great challenge and has undeniable potential regarding future applications.

## Competing interests

The authors declare that they have no competing interests.

## Authors' contributions

RRR and AA wrote the manuscript. The authors read and approved the final manuscript.
